# Media amplification, model source cues, and expectancy violation in public acceptance of generative AI: evidence from a health-consultation experiment

**DOI:** 10.3389/fpsyg.2026.1902291

**Published:** 2026-08-26

**Authors:** Zhiyue Liu, Zhongchao Zhang

**Affiliations:** School of Journalism and Communication, Shandong University, Jinan, China

**Keywords:** behavioral intention, expectancy violations theory, generative AI, perceived risk, social amplification of risk framework, source credibility

## Abstract

**Background:**

Generative artificial intelligence (GenAI) is increasingly used for health-information seeking, where evaluations may depend on prior media amplification, the model identity presented, and expectancy violation.

**Methods:**

We conducted a 2 (media amplification: benefit vs. risk) × 2 (model source: general-purpose vs. professional) × 2 (expectancy violation: positive vs. negative) between-subjects online experiment with 491 participants. Participants read prior-user reports of better- or worse-than-expected performance without using the AI or observing a response. Type III factorial models tested attitude toward use (ATT), behavioral intention (BI), and perceived risk (PRISK). A secondary analysis estimated conditional indirect associations between expectancy violation and BI through PRISK.

**Results:**

Media amplification increased PRISK but did not change ATT or BI on average. Professional model-source cues and positive expectancy violation produced favorable average effects across all three outcomes. In the primary Type III models, the three-way interactions reached significance for ATT, BI, and PRISK (partial η^2^ = 0.008–0.009). Sensitivity support was strongest for the PRISK interaction; the ATT and BI terms varied across specifications. Conditional indirect associations between expectancy violation and BI through lower PRISK were observed in three of the four conditions, while the joint-moderation index included zero.

**Conclusion:**

In this health-consultation vignette, media amplification most clearly altered perceived risk, while model source and expectancy violation shaped prospective acceptance across communication conditions. The three-way evidence was most consistent for perceived risk.

## 1 Introduction

Generative artificial intelligence (GenAI) has become part of routine information seeking and decision support. Human-machine communication research treats AI systems as communicative sources that people evaluate and respond to (Guzman and Lewis, 2020; Lee, 2024). Users often encounter GenAI through media accounts, product descriptions, interface cues, and reports from other users, which can establish expectations before substantive quality is evaluated (Yang and Sundar, 2024).

Health-information consultation requires dedicated analysis because it differs from writing, entertainment, and other low-consequence uses of GenAI. A fluent but incomplete response may omit warning signs, provide inappropriate reassurance, delay professional care, or encourage reliance beyond the system’s competence. Ordinary users may also struggle to assess clinical completeness or recognize consequential omissions and qualifications. The central evaluation problem is whether reliance is appropriate for a particular health-information task under conditions of asymmetric expertise, uncertainty, and possible harm (Longoni et al., 2019; Dunn et al., 2023; Meskó and Topol, 2023).

AI-supported health-information seeking increasingly places conversational systems in the role of accessible advisory sources (Link and Beckmann, 2025; Love et al., 2026). Comparisons of chatbot and physician responses show why users may find AI-generated health communication helpful or empathic, while also underscoring unresolved questions of reliability and accountability (Ayers et al., 2023). Health-communication scholarship accordingly emphasizes uncertainty communication, oversight, professional responsibility, and the conditions under which lay audiences treat AI-generated advice as authoritative (Romero-Rodriguez and Castillo-Abdul, 2025). These features limit the transferability of findings from general technology adoption to health consultation.

Users nevertheless evaluate health-oriented GenAI within a communication setting before they can verify its output. Media amplification may foreground convenience and practical value or, conversely, unreliable answers, overlooked warning signs, and the hazards of overreliance. A general-purpose or professional model label supplies a further cue about domain competence. Positive or negative expectancy violation was operationalized through reports that prior users judged the system as performing better or worse than expected. These manipulated factors are analytically distinct, but their effects may be interdependent because each changes the meaning and diagnostic value of the others.

Existing studies identify usefulness, trust, privacy concern, and perceived risk as central to conversational-AI evaluation (Leschanowsky et al., 2024; Ng and Zhang, 2025). Research on automated decision-making further shows that perceived usefulness, fairness, and risk vary across media, public-health, and judicial contexts (Araujo et al., 2020). Research on collaborative human-GenAI decision-making also shows that acceptance depends on task context and the configuration of AI participation (Hao et al., 2024). This literature leaves open why the same professional label or favorable report carries different weight across communication settings. For health-related GenAI, acceptance denotes conditional reliance: a prospective judgment that a particular system is suitable enough for a bounded task under a specific configuration of communication cues.

The study integrates the Social Amplification of Risk Framework (SARF), Source Credibility Theory, and Expectancy Violations Theory (EVT) in a sequence linking mediated salience, provisional judgments of expertise, and appraisal of expectancy-relevant information.

Using a general health-consultation vignette, the study tests the average and interactive effects of media amplification, model source, and expectancy violation on attitude toward use (ATT), behavioral intention (BI), and perceived risk (PRISK) in a 2 × 2 × 2 experiment. A secondary analysis examines the conditional indirect association between expectancy violation and BI through PRISK and whether that association varies across amplification and source conditions. The design thereby connects mediated risk salience, source-based expertise judgments, and expectancy violation within a common experimental framework.

## 2 Theoretical framework and hypotheses

### 2.1 Expectancy violations theory and the valence of expectancy violation

Expectancy Violations Theory (EVT) explains how people reassess an interaction when behavior deviates from what they anticipated. In Burgoon’s work on interpersonal distance, nonverbal behavior, and interaction norms, people enter communication with expectations shaped by social rules, role identities, and contextual cues (Burgoon and Jones, 1976; Burgoon, 1978; Burgoon and Hale, 1988). When those expectations are violated, attention increases and the meaning of the deviation becomes central to later evaluation (Burgoon and Walther, 1990; Burgoon, 1993). The key issue is whether the behavior is interpreted as better or worse than expected (Burgoon, 2015).

Three EVT concepts are especially relevant here. Expectation refers to generalized norms and more specific situational or role-based assumptions (Burgoon, 1993, 2015). Violation valence refers to whether the deviation is evaluated positively or negatively and how strongly it is judged (Burgoon and Walther, 1990). Reward valence concerns whether the actor is perceived as competent, attractive, or otherwise valuable enough that ambiguous violations may be interpreted more favorably (Burgoon, 1993). These concepts show that violation effects depend on valence: a positive deviation can improve evaluation, whereas a negative deviation can reduce trust, satisfaction, and willingness to continue interaction (Burgoon, 2015; Rheu et al., 2024).

Positive and negative expectancy violations can have asymmetric effects. Positive disconfirmation can increase satisfaction and continuance when performance exceeds an established standard (Oliver, 1980; Bhattacherjee, 2001). Negative discrepancies may be more diagnostic in consequential decisions because negative information often receives greater evaluative weight and signals possible loss, unreliability, or norm breach (Rozin and Royzman, 2001). Algorithm-evaluation research likewise shows both algorithm appreciation and aversion: people may prefer algorithmic judgment, yet observed errors can produce disproportionate withdrawal, with the balance varying across tasks and performance conditions (Dietvorst et al., 2015; Logg et al., 2019; Mahmud et al., 2022; Buder et al., 2024).

This logic transfers to GenAI, where product claims, media reports, interface design, prior experience, and assumptions about AI capability shape expectations before use (Yang and Sundar, 2024). In this experiment, expectancy violation was operationalized through a prior-user report describing performance as clearer, more cautious, or more reliable than expected, or as vague, inconsistent, and below expectations. The report supplied expectancy-relevant information for participants’ prospective evaluation of the system.

A related comparison process appears in post-adoption research. Consumer satisfaction research emphasizes the comparison between prior expectations and direct experience (Oliver, 1980), while the expectation-confirmation model links continued use to confirmation and perceived usefulness (Bhattacherjee, 2001). EVT adds a communicative layer by treating deviations from expectation as socially interpreted events that prompt attention and reassessment of the interaction partner (Burgoon, 1993, 2015). The present pre-use design examines communicated evidence about other users’ assessments, whereas post-adoption confirmation concerns users’ direct experience.

Expectancy violation may be especially salient in digital and AI-mediated environments, where users often infer system quality from limited cues before use. In computer-mediated communication, sparse cues can intensify selective perception and idealized inference (Walther, 1996). In GenAI contexts, marketing discourse, social hype, and fluent output can all raise expectations (Ng and Zhang, 2025). Under these conditions, the difference between competent performance and better-than-expected performance becomes analytically important, as does the difference between ordinary mistakes and performance that falls below a reasonable standard.

Prior studies of chatbots and embodied agents show that expectancy violation can affect perceived helpfulness, competence, trust, and willingness to continue interaction (Burgoon et al., 2016; Rheu et al., 2024). This evidence places expectancy violation within the expectations established by source cues and the wider communicative context. In GenAI research, positive and negative violations are likely to have different consequences depending on the model’s perceived identity and the environment in which the interaction occurs.

Recent human-machine communication (HMC) scholarship strengthens this point by treating AI as a communicative source whose perceived agency, expertise, and accountability shape user response (Guzman and Lewis, 2020; Lee, 2024). GenAI makes the source question difficult because authorship can be ambiguous: users may respond to the model, the interface, the institution behind it, or the composite identity created by all three. Work on machine heuristics and explainable AI similarly indicates that users rely on simplified source and capability cues when they cannot inspect the system’s reasoning (Xu and Shi, 2024; Yang and Sundar, 2024). Expectations in human-AI interaction are assembled before the focal performance information appears.

Expectancy violation is therefore a context-dependent appraisal of better- or worse-than-expected information. Chatbot research shows that disappointment can reshape competence judgments and future engagement, while research on computer agents indicates that responses to algorithmic actors vary across tasks and performance conditions (Buder et al., 2024; Rheu et al., 2024). In health consultation, negative reports may be especially consequential because they implicate missed warning signs or inappropriate reassurance. Favorable reports gain persuasive force when users can reconcile them with the model’s claimed domain identity and the evidentiary standard established by the preceding media-amplification condition.

Health-consultation settings make these processes salient because uncertainty is high and errors can matter. Positive expectancy violation may improve evaluation and reduce anticipated danger, whereas negative expectancy violation may lead users to question both usefulness and the appropriateness of reliance (Rheu et al., 2024).

H1a-H1c: Compared with negative expectancy violation, positive expectancy violation will increase ATT and BI and reduce PRISK, averaged across media-amplification and model-source conditions.

### 2.2 Social amplification of risk framework and media amplification

The Social Amplification of Risk Framework (SARF) explains why some hazards generate public reactions that exceed their immediate material consequences, while others attract muted responses despite objective seriousness (Kasperson et al., 1988). Its central point is that people interpret risk through social and communicative processes. Risk signals move through amplification stations such as expert institutions, news media, political discourse, and interpersonal networks, where they are selected, interpreted, and retransmitted. As a result, some dangers become more salient, emotionally charged, morally framed, or socially consequential than others (Kasperson et al., 1988; Kasperson et al., 2022). Amplification can affect what people fear as well as how they interpret uncertainty, responsibility, and acceptable action (Bearth and Siegrist, 2022).

Amplification processes are particularly visible in digital media environments, where repetition, emotional framing, and platform visibility can concentrate attention on selected aspects of a technology. Media coverage and public discourse about GenAI often move between celebration and caution. GenAI is portrayed as efficient and transformative, but it is also framed as a source of hallucination, bias, privacy leakage, and misinformation (Deng and Ahmed, 2025; Zhang and Pang, 2026). These messages supply interpretive schemes that users carry into later evaluations.

Drawing on SARF, the media-amplification manipulation uses an experimentally abstracted stimulus design based on the framework’s account of signal selection and emphasis. The two conditions capture this selected component of SARF while preserving the controlled factorial structure of the experiment. Benefit amplification foregrounds convenience and practical value, whereas risk amplification foregrounds inaccurate answers, missed warning signs, and the harms of overreliance. Accordingly, the condition labels refer to SARF-informed experimental abstractions rather than a complete operationalization of the amplification process.

These dimensions are directly relevant to general health-information consultation. The benefit emphasis captures common reasons for seeking conversational assistance, such as organizing information and preparing questions, whereas the risk emphasis captures communication failures that can matter even when the system is not used for diagnosis or treatment. Holding the task and vignette structure constant while varying these emphases permits a focused comparison of opportunity and harm salience.

Many users encounter GenAI through mediated discourse before direct use. Media reports, platform communication, social media discussion, and institutional warnings all help set expectations (Deng and Ahmed, 2025). A helpful and reliable portrayal may make users more likely to notice advantages and interpret competent performance as confirmation or pleasant surprise. A portrayal centered on unsafe, misleading, or hard-to-regulate systems may increase attention to errors, motivate verification, and raise evaluative standards (Kasperson et al., 1988; Bearth and Siegrist, 2022).

Recent AI-risk research supports this extension of SARF. Analyses of AI risk frames on digital platforms show that public discourse selects the dangers, benefits, and governance problems through which later evaluations are made (Schwarz, 2024). Work on expert dynamics in risk amplification further shows that attenuation and amplification begin before messages reach lay audiences, as expert claims, institutional authority, and media selection help determine which risks become visible (Fjaeran et al., 2024). In AI contexts, risk amplification may increase fear, but it can also increase demand for credible source cues, traceable evidence, and visible accountability.

Empirical studies of AI-related risk information also show that amplified risk information can move users toward protective or avoidance-oriented responses, while uncertainty about AI chatbot information can motivate further risk information seeking (Hong, 2025; Zhang and Pang, 2026). This evidence suggests that risk amplification can raise the evidentiary threshold for acceptance by directing attention to whether a specific AI source is professionally appropriate and whether performance information adequately addresses perceived harm.

From the social-amplification perspective, media amplification may affect both average evaluation and the interpretation of later cues. Benefit amplification may support favorable attitudes and intentions, whereas risk amplification should most directly increase PRISK by making error consequences and uncertainty salient. Media amplification may also change the diagnostic value of model source and expectancy violation. These main-effect predictions are averages across the other randomized factors.

This point is especially relevant to health consultation. Risk cues in this setting implicate possible harm, delayed treatment, privacy breaches, and responsibility for adverse outcomes. A risk-amplification condition may raise the threshold for trust, making users more sensitive to negative expectancy violation and less easily reassured by favorable performance information. Benefit-amplification conditions may make users more receptive to positive expectancy violation and more willing to translate favorable information into adoption intention.

*H2a–c*: Compared with benefit amplification, risk amplification will reduce ATT and BI and increase PRISK, averaged across model-source and expectancy-violation conditions.

*H3a–c*: Media amplification will moderate the effect of expectancy violation on ATT, BI, and PRISK.

### 2.3 Source credibility theory and model-source credibility

As GenAI becomes common in information seeking and decision support, users increasingly treat conversational systems as information sources. Their outputs can appear fluent and confident despite unclear authorship, attribution, and evidentiary provenance. Users must therefore assess whether the system is credible enough to rely on.

Source credibility theory offers a useful framework for this problem. Classic persuasion research argues that message acceptance depends on message content and on the audience’s assessment of source credibility, especially expertise and trustworthiness (Hovland and Weiss, 1951; Hovland et al., 1953). Later work distinguishes dimensions such as competence, trustworthiness, and goodwill and develops scales for measuring them (McCroskey, 1966; McCroskey and Teven, 1999; Ohanian, 1990). Source cues matter most when users cannot easily verify information directly. Under uncertainty, limited motivation, or limited ability, audiences often use identity labels, institutional associations, and other heuristics to evaluate message content (Metzger et al., 2003; Metzger et al., 2010; Pornpitakpan, 2004).

GenAI presents a composite source that includes the conversational agent, the platform behind it, the model’s claimed training basis, the product’s professional framing, and interface cues about evidence or uncertainty. Its credibility is formed through technical performance, product positioning, institutional backing, and visible communicative design (Yang and Sundar, 2024).

This composite-source problem is acute in medicine. Most ordinary users cannot independently assess whether a health-related AI response is medically sound, reflects domain expertise, or omits important boundary conditions. They may rely on identity cues that suggest professional competence. A professional medical model cue can imply stronger domain knowledge, better judgment, and closer task fit. A general-purpose conversational model may be seen as broad and useful for general discussion but less dependable for specialized medical questions (Lee and See, 2004; Lim and Schmälzle, 2024).

In practice, professionalism is communicated through product positioning, domain-specific evidence, uncertainty markers, and referral guidance. As an experimental manipulation, the general-versus-professional distinction captures a source-credibility shortcut: whether users perceive the AI as possessing superior knowledge, skills, and judgment in the relevant domain. Research on source disclosure and machine heuristics supports the idea that users rely on such markers when evaluating AI-generated messages under uncertainty (Lim and Schmälzle, 2024; Yang and Sundar, 2024).

Recent HMC and health-chatbot studies show that AI source credibility is produced through multiple design and identity cues. Source disclosure can change evaluations of AI-generated messages, and health-chatbot status cues and message contingency can affect credibility assessment (Dong and Wu, 2025; Lim and Schmälzle, 2024). Experiments on virtual physicians also show that chatbot design cues and threat perception jointly influence health-information trust, while work on personalized and careful AI-generated health advice indicates that trust and adoption depend on communicated caution and task fit (Jin and Eastin, 2025; Qin et al., 2024). Public resistance to health chatbots appears to involve perceived usefulness, perceived risk, and trust-related mechanisms, which further links source cues to risk appraisal (Zou et al., 2024).

A professional model cue supports an initial credibility inference whose force depends on whether media amplification makes expertise consequential and whether the prior-user report is consistent with the competence implied by the label. In Source Credibility Theory, the cue supplies a heuristic inference of expertise; in EVT, that inference contributes to communicator reward valence and may alter appraisal of favorable or unfavorable expectancy violation.

The connection yields a directional interaction prediction. A professional label should make a favorable report of better-than-expected performance more diagnostic for ATT and BI and more reassuring for PRISK than the same report attached to a general-purpose model. Conversely, worse-than-expected information may erode the advantage implied by a professional label. Media amplification should further condition the professional-source advantage because domain fit becomes especially relevant when risk amplification makes error and overreliance salient.

The health-consultation context makes these mechanisms especially visible. Because the perceived cost of error is high, users may look for stronger expertise cues before relying on a system. Professional-source cues are expected to increase acceptance and reduce perceived risk, while also changing how users interpret positive and negative expectancy violation.

*H4a–c*: Compared with a general-purpose model, a professional model-source cue will increase ATT and BI and reduce PRISK, averaged across media-amplification and expectancy-violation conditions.

*H5a–c*: The favorable effect of positive versus negative expectancy violation will be larger for a professional than for a general-purpose model for ATT and BI and will produce a larger reduction in PRISK.

*H6a–c*: The professional-versus-general model advantage will be larger under risk amplification than under benefit amplification for ATT and BI and will produce a larger reduction in PRISK.

### 2.4 Integrating SARF, source credibility theory, and EVT

The three theoretical perspectives address connected links in a single communication sequence. SARF explains how media amplification makes benefits or risks salient before a specific system is evaluated. Source Credibility Theory explains how a professional model label supports a provisional expertise judgment when health information cannot be verified directly. EVT explains how better- or worse-than-expected information is evaluated in relation to the communicator’s reward valence. Their integration links mediated salience, source judgment, and evaluation following expectancy violation in one sequence.

The sequence also corresponds to the order of the experimental cues. Benefit or risk amplification first establishes an interpretive context by foregrounding opportunity or harm. The model-source cue then identifies the kind of competence being claimed: a professional model implies domain specialization, whereas a general-purpose model implies broad but less task-specific capability. Finally, a prior-user report indicates that the system was evaluated as performing better or worse than expected.

Evidence shows that trust in AI varies with performance, task demands, and user context (Glikson and Woolley, 2020; Kaplan et al., 2023). Users may reject or prefer algorithmic advice depending on the task’s demands for expertise, empathy, or accountable judgment (Castelo et al., 2019; Dietvorst et al., 2015; Logg et al., 2019; Mahmud et al., 2022; Yalcin et al., 2022). Resistance to medical AI is especially likely when consumers believe that an algorithm cannot account for the uniqueness of their condition, even when favorable performance information is available (Longoni et al., 2019). Health consultation is thus theoretically informative because it combines accessibility with a salient possibility of harm.

The link between Source Credibility Theory and EVT is particularly important. In EVT, communicator reward valence affects how a violation is interpreted. In the present setting, model identity supplies one basis for that reward value because a professional model may be judged more capable of producing appropriate health information. A favorable prior-user report may therefore be more diagnostic when it is consistent with a credible claim of domain competence, whereas a negative report may erode the advantage implied by that claim. Source credibility thus helps explain why the same expectancy-violation report can carry different weight across model identities.

The higher-order prediction follows from the proposed joint operation of the three factors. The meaning of a professional label and a favorable or unfavorable report should depend on whether the media-amplification condition foregrounds benefits or risks. The three-way term therefore tests whether the effects of model source and expectancy violation vary with the media-amplification condition. Because the lower-order terms average across the third factor, they are read together with the condition-specific estimates when the three-way term is significant.

Perceived risk connects the communication sequence to judgments of appropriate reliance. Media amplification affects which harms are salient, model source affects whether the system appears capable of managing those harms, and expectancy violation supplies information relevant to revising that judgment. Users may evaluate a system favorably while remaining unwilling to rely on it if anticipated harm and uncertainty remain high. PRISK is therefore both a theoretically relevant outcome and a plausible concurrent statistical pathway.

*H7a–c*: Media amplification, model source, and expectancy violation will jointly interact to shape ATT, BI, and PRISK.

### 2.5 Perceived risk and conditional indirect associations

The factorial hypotheses establish whether randomized communication cues change ATT, BI, and PRISK. A related question is whether expectancy violation is conditionally associated with BI through perceived risk. In health consultation, a favorable prior-user report may coincide with lower anticipated harm and stronger willingness to use the system, whereas an unfavorable report may coincide with greater uncertainty and weaker intention.

Expectation-confirmation research links continuance intention to changes in usefulness, reliability, and satisfaction after experience (Oliver, 1980; Bhattacherjee, 2001). EVT adds that better- or worse-than-expected information can prompt reassessment of the interaction partner (Burgoon, 1993, 2015). Trust-in-automation and source-credibility research further indicates that reliance depends on whether a system appears safe, manageable, and appropriate for the task when direct verification is difficult (Lee and See, 2004; Metzger et al., 2010; Lim and Schmälzle, 2024; Ng and Zhang, 2025). Together, these perspectives support examining whether the randomized expectancy contrast is related to BI through differences in PRISK.

Because PRISK and BI were measured in the same post-exposure questionnaire, the secondary analysis estimates their conditional indirect association.

The association may differ across media-amplification and model-source conditions. Risk amplification foregrounds possible harm, while a professional model source signals task-relevant competence. The strength of the indirect association between positive expectancy violation and stronger intention through lower PRISK may therefore vary across these configurations. H8b is evaluated from the omnibus three-way index of the indirect association.

*H8a*: Compared with negative expectancy violation, positive expectancy violation will be conditionally indirectly associated with stronger BI through lower PRISK.

*H8b*: Media amplification and model source will jointly moderate the conditional indirect association of expectancy violation with BI through PRISK.

## 3 Materials and methods

### 3.1 Method

#### 3.1.1 Research design

This study used a 2 (media amplification: benefit vs. risk) × 2 (model source: general-purpose vs. professional) × 2 (expectancy violation: positive vs. negative) between-subjects online experiment. The study focused on general health-information consultation. Participants evaluated written vignettes containing prior-user reports that described better- or worse-than-expected performance; they did not interact with an AI system or observe an AI-generated response.

#### 3.1.2 Procedure and stimulus materials

The online experiment was fielded in December 2025. Participants were recruited through Credamo, a Chinese online research platform (*n* = 354), social media (*n* = 94), and university or community networks (*n* = 43). They first completed demographic and pre-exposure measures and were then randomly assigned to one of eight stimulus conditions. After reading the assigned vignette, participants completed the post-stimulus outcomes, manipulation checks, and data-quality items.

The eight stimuli were developed as parallel vignettes with three components presented in a fixed order: a media-amplification stimulus, a model-source description, and a prior-user evaluation. Consistent with the SARF-informed experimental abstraction described in Section 2.2, the media-amplification component varied the selection and emphasis of benefit- and risk-related signals. Benefit amplification emphasized convenience, efficiency, and practical value because these are prominent reasons for using conversational systems to obtain or organize health information. Risk amplification emphasized inaccurate answers, missed warning signs, and harms from overreliance because these are consequential risks in health-information consultation. The manipulation contrasted the salience of task-relevant opportunities and harms while keeping the communication domain constant.

The professional-model condition described DocAI as designed and trained specifically for medical consultation; the general-purpose condition described ChatAI as a broad conversational system designed for general use. Positive expectancy violation was operationalized through a prior-user report stating that the system was clearer, more cautious, or more reliable than expected. Negative expectancy violation was operationalized through a report of vague or inconsistent explanations, omitted warnings, or performance below expectations. The health-consultation task, presentation order, approximate length, and nonfocal information were held as constant as practicable across conditions.

An independent pretest used the same 2 × 2 × 2 stimulus structure. After quality screening, the analyzable sample was *N* = 160, with 20 participants per cell. Six directional items assessed perceived benefit and risk emphasis, professional and general model identity, and positive and negative expectancy violation. Participants also evaluated the clarity, realism, and readability of the assigned vignette on seven-point scales. Pretest participants were not included in the main experiment.

#### 3.1.3 Participants

A total of 528 responses were collected. The stated quality-control criteria excluded 37 responses: nine for short completion time, nine for failed attention checks, seven for comprehension errors, six for straightlining or low response variance, and six for duplicate IP addresses combined with abnormal response patterns. The final analysis sample was *N* = 491 (93.0% retained), with experimental cells ranging from 60 to 63 participants. Participants ranged from 18 to 56 years of age (*M* = 31.43, SD = 8.18); 236 (48.1%) were men, 247 (50.3%) were women, and 8 (1.6%) did not disclose a binary gender. More than half held a bachelor’s degree (*n* = 275, 56.0%), followed by associate degrees (*n* = 108, 22.0%), master’s degrees or above (*n* = 69, 14.1%), and high school education or below (*n* = 39, 7.9%); 112 participants (22.8%) reported a chronic health condition. The prior GenAI-use-frequency score averaged 3.70 (SD = 1.09), self-rated AI-use skill averaged.

3.89 (SD = 1.02), and 455 participants reported some prior use of AI for health information.

Measured pre-exposure characteristics did not differ significantly across the eight conditions (all *p* ≥ 0.21). Randomized factorial models without covariates were retained as the primary analyses, with covariate-adjusted models used as robustness checks.

#### 3.1.4 Measures

The questionnaire included pre-exposure measures, focal post-stimulus outcomes, manipulation checks, and data-quality items. Unless otherwise noted, multi-item constructs used scales ranging from 1 = strongly disagree to 7 = strongly agree. Item wording was adapted to the focal AI system and the health-consultation task. Scale scores were calculated by averaging the relevant items after reverse-worded items were recoded. The full questionnaire, response options, and reverse-coding information are provided in Supplementary Material, Section S2.

Attitude toward use (ATT) was measured with two items adapted from Davis (1989) and Taylor and Todd (1995), assessing participants’ overall evaluation of using the focal AI for health consultation and whether such use was a good idea. Behavioral intention (BI) was measured with two items adapted from Davis (1989) and Venkatesh et al. (2003), assessing future-use intention and willingness to use the focal AI regularly for health information. ATT and BI were specified as narrow, unidimensional constructs, and the two representative items for each construct were evaluated using coefficient alpha, confirmatory factor loadings, composite reliability (CR), and average variance extracted (AVE).

Perceived risk (PRISK) was measured with three items grounded in research on risk amplification, AI risk perception, and uncertainty in AI-assisted information seeking (Kasperson et al., 1988; Bearth and Siegrist, 2022; Klein et al., 2024; Hong, 2025). The items assessed potential harm, erroneous decisions, and non-negligible uncertainty.

Pre-exposure measures used for sample description, randomization assessment, and sensitivity models are specified in Section 3.1.6.

Post-stimulus checks assessed perceived amplification, model source, and expectancy violation. Reading, comprehension, and directed-attention items supported quality screening. The focal post-stimulus constructs showed satisfactory reliability and convergent validity. Coefficient alpha was 0.826 for ATT, 0.963 for BI, and 0.951 for PRISK. A three-factor confirmatory factor analysis (CFA) of these constructs showed good fit, χ^2^ (11) = 5.28, *p* = 0.917, comparative fit index (CFI) = 1.000, Tucker-Lewis index (TLI) = 1.000, root mean square error of approximation (RMSEA) = 0.000, and standardized root mean square residual (SRMR) = 0.013. Standardized loadings ranged from 0.761 to 0.924 for ATT, 0.933 to 0.995 for BI, and 0.915 to 0.944 for PRISK. CR ranged from 0.833 to 0.964 and AVE from 0.716 to 0.930. Table 1 reports the construct-specific estimates.

**Table 1:** Reliability and convergent-validity evidence for focal post-stimulus constructs (*N* = 491).

Construct	Items	Coefficient α	Loading range	CR	AVE
ATT	2	0.826	0.761–0.924	0.833	0.716
BI	2	0.963	0.933–0.995	0.964	0.930
PRISK	3	0.951	0.915–0.944	0.952	0.868

ATT, Attitude toward use; BI, Behavioral intention; PRISK, Perceived risk; CR, Composite reliability; AVE, Average variance extracted. Loading ranges are standardized CFA loadings.

#### 3.1.5 Stimulus pretest

Pretest contrasts were evaluated with Welch-corrected independent-samples t tests and Holm adjustment across the six directional checks. All contrasts were in the intended direction. Benefit-amplification perception was higher in the benefit condition than in the risk condition (*M* = 4.84, SD = 0.77 vs. *M* = 4.06, SD = 0.92, d = 0.914, Holm-adjusted *p* < 0.001), and risk-amplification perception was higher in the risk condition than in the benefit condition (*M* = 4.78, SD = 0.90 vs. *M* = 4.10, SD = 0.74, d = 0.829, *p* < 0.001). Professional-model perception was higher under the professional than the general-purpose cue (*M* = 4.78, SD = 0.79 vs. *M* = 4.05, SD = 0.87, d = 0.880, *p* < 0.001), whereas general-model perception was higher under the general-purpose cue (*M* = 4.62, SD = 0.88 vs. *M* = 4.24, SD = 0.89, d = 0.439, *p* = 0.006).

Positive expectancy-violation perception was higher after the positive than the negative prior-user report (*M* = 4.84, SD = 0.74 vs. *M* = 4.26, SD = 0.69, d = 0.810, Holm-adjusted *p* < 0.001), and negative expectancy-violation perception was higher after the negative report (*M* = 4.61, SD = 0.79 vs. *M* = 4.08, SD = 0.73, d = 0.697, *p* < 0.001). Vignette quality ratings were favorable: clarity averaged 5.67 [SD = 0.81, 95% confidence interval (CI) (5.55, 5.80)], realism averaged 5.49 [SD = 0.91, 95% CI (5.35, 5.63)], and readability averaged 5.79 [SD = 0.59, 95% CI (5.70, 5.89)]. The pretest therefore supports recognition of all three manipulations while indicating that the materials remained clear, realistic, and readable.

#### 3.1.6 Analytic strategy

ATT, BI, and PRISK were analyzed with separate 2 × 2 × 2 factorial models. Because cell sizes were slightly unequal, primary analyses used Type III sums of squares with sum contrasts. Main effects and two-way interactions were interpreted as averages across the remaining factor and were qualified by condition-specific estimates whenever the three-way term was significant. Effect sizes are reported as partial η^2^. Estimated marginal means and simple effects were used to describe significant interactions. Heteroskedasticity-consistent type 3 (HC3) tests and models adjusted for measured pre-exposure characteristics served as sensitivity analyses. The covariate-adjusted models included age, gender, education, chronic-condition status, self-rated health, recent consultation need, prior GenAI-use frequency, self-rated AI-use skill, prior use of AI for health information, recruitment source, general AI attitude, risk aversion, health involvement, and pre-existing expectations. Ordinal covariates were entered using their numeric codes; gender, education, and recruitment source were indicator-coded. Manipulation checks and other post-treatment variables were not entered as covariates.

The conditional indirect-association analysis used the same sample. Both equations were estimated by ordinary least squares. The PRISK and BI equations included media amplification, model source, expectancy violation, all two-way interactions, and the three-way interaction; the BI equation additionally included PRISK. The primary factorial models used sum contrasts, whereas the conditional indirect-association equations used 0/1 dummy coding: media amplification was coded 0 = benefit and 1 = risk, model source was coded 0 = general-purpose and 1 = professional, and expectancy violation was coded 0 = negative and 1 = positive. The benefit/general-purpose/negative condition served as the reference category. Because the two analyses use different parameterizations, their individual coefficients are not directly comparable. The specification reflects the study’s theoretical focus on how media amplification and model source condition risk appraisal following expectancy-related information. Accordingly, the PRISK-to-BI coefficient was held constant across the four media-amplification × model-source configurations, and the conditional products varied through the condition-specific expectancy-violation-to-PRISK coefficients. PRISK interactions with the two moderators were not included. Four condition-specific products were calculated from the relevant expectancy-violation coefficients in the PRISK equation and the constant PRISK-to-BI coefficient. Percentile confidence intervals for the products, all six pairwise contrasts, and the corresponding simple and omnibus indices were estimated from 5,000 bootstrap samples resampled within experimental cells. H8b was evaluated from the confidence interval for the omnibus three-way index.

## 4 Results

The primary analyses used the final sample of *N* = 491 and an error degree of freedom of 483. The three-factor CFA fit the data well, and ATT, BI, and PRISK showed satisfactory reliability and convergent validity (Table 1). Media amplification had its largest average effect on PRISK (partial *η*^2^ = 0.195), while model source and expectancy violation showed significant average effects on ATT, BI, and PRISK. The three-way interactions were significant for all three outcomes (partial *η*^2^ = 0.008–0.009). Full factorial results are reported in Table 2.

**Table 2:** Factorial-model results for ATT, BI, and PRISK (*N* = 491).

Outcome	Effect	*F* (1, 483)	*p*	Partial *η*^2^	HC3 *p*
ATT	Media amplification	0.01	= 0.921	0.000	= 0.921
ATT	Model source	27.16	< 0.001	0.053	< 0.001
ATT	Expectancy violation	49.67	< 0.001	0.093	< 0.001
ATT	Media amplification × model source	7.05	= 0.008	0.014	= 0.009
ATT	Media amplification × expectancy violation	6.42	= 0.012	0.013	= 0.012
ATT	Model source × expectancy violation	10.97	= 0.001	0.022	= 0.001
ATT	Media amplification × model source × expectancy violation	4.48	= 0.035	0.009	= 0.036
BI	Media amplification	0.12	= 0.728	0.000	= 0.730
BI	Model source	14.59	< 0.001	0.029	< 0.001
BI	Expectancy violation	26.00	< 0.001	0.051	< 0.001
BI	Media amplification × model source	0.12	= 0.727	0.000	= 0.729
BI	Media amplification × expectancy violation	4.53	= 0.034	0.009	= 0.035
BI	Model source × expectancy violation	6.26	= 0.013	0.013	= 0.013
BI	Media amplification × model source × expectancy violation	3.91	= 0.049	0.008	= 0.050
PRISK	Media amplification	117.26	< 0.001	0.195	< 0.001
PRISK	Model source	24.01	< 0.001	0.047	< 0.001
PRISK	Expectancy violation	35.33	< 0.001	0.068	< 0.001
PRISK	Media amplification × model source	0.21	= 0.645	0.000	= 0.648
PRISK	Media amplification × expectancy violation	3.39	= 0.066	0.007	= 0.069
PRISK	Model source × expectancy violation	0.18	= 0.674	0.000	= 0.677
PRISK	Media amplification × model source × expectancy violation	4.43	= 0.036	0.009	= 0.037

ATT, Attitude toward use; BI, Behavioral intention; PRISK, Perceived risk. Tests are based on Type III factorial models with sum contrasts. HC3 p values are heteroskedasticity-robust sensitivity-test p values. Partial η^2^ denotes partial eta-squared. All tests use df = 1, 483.

### 4.1 Attitude toward use

For ATT, the average effects of model source, *F* (1, 483) = 27.16, *p* < 0.001, partial *η*^2^ = 0.053, and expectancy violation, *F* (1, 483) = 49.67, *p* < 0.001, partial *η*^2^ = 0.093, were significant, whereas media amplification was not, *F* (1, 483) = 0.01, *p* = 0.921, partial *η*^2^ < 0.001. Averaged across the remaining factors, professional-model cues and positive expectancy violation were associated with more favorable attitudes. These contrasts were consistent with H1a and H4a at the averaged level; H2a was not supported.

All three ATT two-way terms were significant: media amplification × model source, *F* (1, 483) = 7.05, *p* = 0.008, partial *η*^2^ = 0.014; media amplification × expectancy violation, *F* (1, 483) = 6.42, *p* = 0.012, partial *η*^2^ = 0.013; and model source × expectancy violation, *F* (1, 483) = 10.97, *p* < 0.001, partial *η*^2^ = 0.022. As averaged two-way contrasts, these results were consistent with H3a, H5a, and H6a.

The ATT three-way term was significant, *F* (1, 483) = 4.48, *p* = 0.035, partial *η*^2^ = 0.009, consistent with H7a. The professional-model advantage was reliable under risk amplification with positive expectancy violation (mean difference = 1.21, *p* < 0.001) and was small or nonsignificant in the other three combinations of media amplification and expectancy violation. The highest-rated condition was risk amplification, professional model, and positive expectancy violation (*M* = 5.36). Positive expectancy violation increased ATT in three of the four combinations of media amplification and model source; the benefit-amplification/general-model condition showed a smaller, nonsignificant contrast (mean difference = 0.30, *p* = 0.124).

### 4.2 Behavioral intention

For BI, the average effects of model source, *F* (1, 483) = 14.59, *p* < 0.001, partial *η*^2^ = 0.029, and expectancy violation, *F* (1, 483) = 26.00, *p* < 0.001, partial *η*^2^ = 0.051, were significant, whereas media amplification was not, *F* (1, 483) = 0.12, *p* = 0.728, partial *η*^2^ < 0.001. Professional-model cues and positive expectancy violation were associated with stronger intention on average. These contrasts were consistent with H1b and H4b at the averaged level; H2b was not supported.

The media amplification × expectancy-violation term, *F* (1, 483) = 4.53, *p* = 0.034, partial *η*^2^ = 0.009, and model source × expectancy-violation term, *F* (1, 483) = 6.26, *p* = 0.013, partial *η*^2^ = 0.013, were significant; media amplification × model source was not, *F* (1, 483) = 0.12, *p* = 0.727. The three-way term was significant, *F* (1, 483) = 3.91, *p* = 0.049, partial *η*^2^ = 0.008. Positive expectancy violation increased BI most strongly in the risk-amplification/professional-model condition (mean difference = 1.24, *p* < 0.001), where the professional-model advantage was also largest (mean difference = 0.92, *p* < 0.001) and was effectively absent under risk amplification with negative expectancy violation. The averaged two-way results were consistent with H3b and H5b but not H6b; the three-way result was consistent with H7b.

### 4.3 Perceived risk

For PRISK, media amplification, *F* (1, 483) = 117.26, *p* < 0.001, partial *η*^2^ = 0.195, model source, *F* (1, 483) = 24.01, *p* < 0.001, partial *η*^2^ = 0.047, and expectancy violation, *F* (1, 483) = 35.33, *p* < 0.001, partial *η*^2^ = 0.068, each had a significant average effect. Risk amplification increased PRISK, whereas professional-model cues and positive expectancy violation reduced it. These averaged contrasts were consistent with H1c, H2c, and H4c.

None of the PRISK two-way terms reached significance: media amplification × model source, *F* (1, 483) = 0.21, *p* = 0.645; media amplification × expectancy violation, *F* (1, 483) = 3.39, *p* = 0.066; and model source × expectancy violation, *F* (1, 483) = 0.18, *p* = 0.674. H3c, H5c, and H6c were therefore not supported as averaged two-way interactions.

The PRISK three-way term was significant, *F* (1, 483) = 4.43, *p* = 0.036, partial *η*^2^ = 0.009, consistent with H7c. Risk amplification increased PRISK in every model-by-expectancy configuration (all *p* < 0.001). Positive expectancy violation reduced PRISK in three of four combinations of media amplification and model source, most strongly under risk amplification with a general-purpose model (mean difference = 0.69, *p* < 0.001); the benefit-amplification/general-model contrast was small and nonsignificant (mean difference = 0.12, *p* = 0.439).

**Figure 1:**
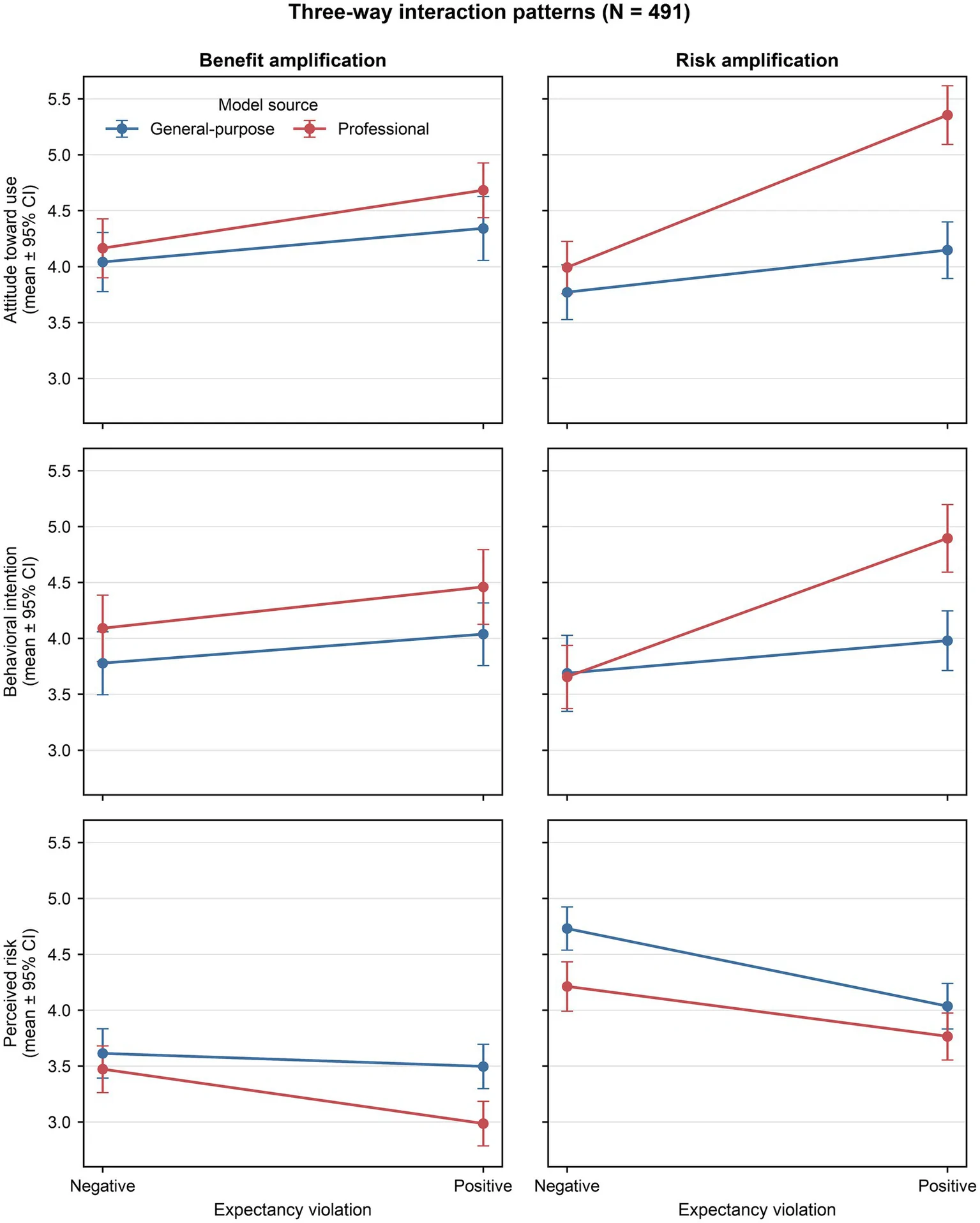
Estimated marginal means and 95% confidence intervals for the three-way interaction among media amplification, model source, and expectancy violation (*N* = 491). The incremental three-way effect sizes were partial *η*^2^ = 0.008–0.009.

Diagnostic checks showed nonsignificant Levene tests for ATT, BI, and PRISK, modest residual skew and excess kurtosis, and maximum Cook’s distances below 0.02. For the three-way terms, the unadjusted HC3 *p* values were 0.0364 for ATT, 0.0501 for BI, and 0.0372 for PRISK; covariate-adjusted ordinary-least-squares p values were 0.0403, 0.0507, and 0.0185, respectively; and covariate-adjusted HC3 p values were 0.0525, 0.0557, and 0.0224. Sensitivity support was therefore strongest for PRISK, while ATT and BI varied across specifications. The estimated marginal means and 95% confidence intervals are displayed in Figure 1.

### 4.4 Conditional indirect-association analysis

**Figure 2:**
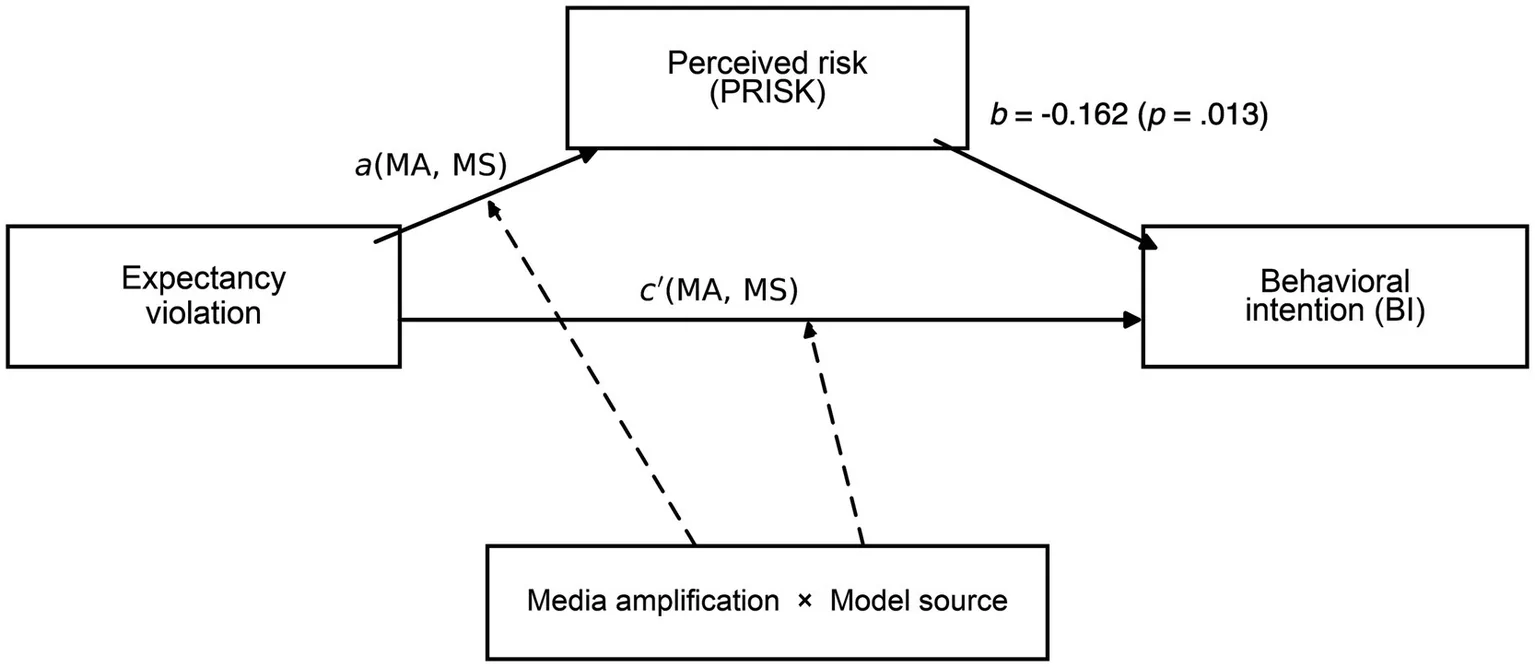
Conditional indirect-association model linking expectancy violation (*X*), perceived risk (*PRISK*), and behavioral intention (*BI*). Expectancy violation was operationalized through prior-user reports; media amplification and model source condition the *X*-to-*PRISK* and direct *X*-to-*BI* associations (*N* = 491).

The secondary analysis tested H8a and H8b using the model specification and bootstrap procedure described in Section 3.1.6. Both equations included the complete factorial terms, with PRISK added to the BI equation; Table 3 reports the coefficients. The PRISK-to-BI coefficient was held constant across the four media-amplification × model-source configurations, so variation among the conditional products came from the condition-specific expectancy-violation-to-PRISK coefficients. Figure 2 summarizes the conditional indirect-association model.

**Table 3:** Full-factorial regression coefficients for the conditional indirect-association analysis (*N* = 491).

Equation	Predictor	*b*	SE	*t*	*p*
PRISK equation	(Intercept)	3.613	0.103	35.04	< 0.001
PRISK equation	MA	1.117	0.146	7.63	< 0.001
PRISK equation	MS	−0.140	0.146	−0.96	= 0.339
PRISK equation	EV	−0.116	0.147	−0.79	= 0.432
PRISK equation	MA × MS	−0.376	0.207	−1.81	= 0.070
PRISK equation	MA × EV	−0.578	0.208	−2.78	= 0.006
PRISK equation	MS × EV	−0.370	0.207	−1.79	= 0.074
PRISK equation	MA × MS × EV	0.617	0.293	2.11	= 0.036
BI equation	(Intercept)	4.364	0.279	15.65	< 0.001
BI equation	PRISK	−0.162	0.065	−2.48	= 0.013
BI equation	MA	0.091	0.223	0.41	= 0.682
BI equation	MS	0.289	0.211	1.37	= 0.170
BI equation	EV	0.241	0.211	1.14	= 0.256
BI equation	MA × MS	−0.406	0.299	−1.36	= 0.176
BI equation	MA × EV	−0.062	0.301	−0.21	= 0.837
BI equation	MS × EV	0.051	0.299	0.17	= 0.865
BI equation	MA × MS × EV	0.938	0.423	2.22	= 0.027

BI, Behavioral intention; PRISK, Perceived risk; MA, Media amplification; MS, Model source; EV, Expectancy violation. MA was coded 0, Benefit amplification and 1, risk amplification; MS was coded 0, general-purpose model and 1, professional model; EV was coded 0, negative expectancy violation and 1, positive expectancy violation. The benefit/general-purpose/negative condition served as the reference category. MA × MS, MA × EV, MS × EV, and MA × MS × EV are products of the corresponding indicator variables. The primary factorial models in Table 2 used sum contrasts, so individual coefficients are not directly comparable across Tables 2, 3. PRISK and BI were measured concurrently, and bootstrap intervals use 5,000 within-cell resamples.

In the PRISK equation, the expectancy-violation contrast was nonsignificant in the benefit-amplification/general-model reference condition, *b* = −0.116, standard error (SE) = 0.147, *t* = −0.79, *p* = 0.432, 95% CI [−0.404, 0.172]. Risk amplification predicted higher PRISK, *b* = 1.117, *p* < 0.001, whereas the model-source coefficient was nonsignificant in the complete factorial specification, *b* = −0.140, *p* = 0.339. The media amplification × expectancy-violation term, *b* = −0.578, *p* = 0.006, and the three-way term, *b* = 0.617, *p* = 0.036, were significant; the other two-way terms were not. These coefficients show that the expectancy-violation association with PRISK varied across conditions.

In the BI equation, PRISK was negatively associated with BI while the complete factorial set was controlled, *b* = −0.162, SE = 0.065, *t* = −2.48, *p* = 0.013, 95% CI [−0.290, −0.034]. The equation also retained a significant three-way term, *b* = 0.938, *p* = 0.027.

**Figure 3:**
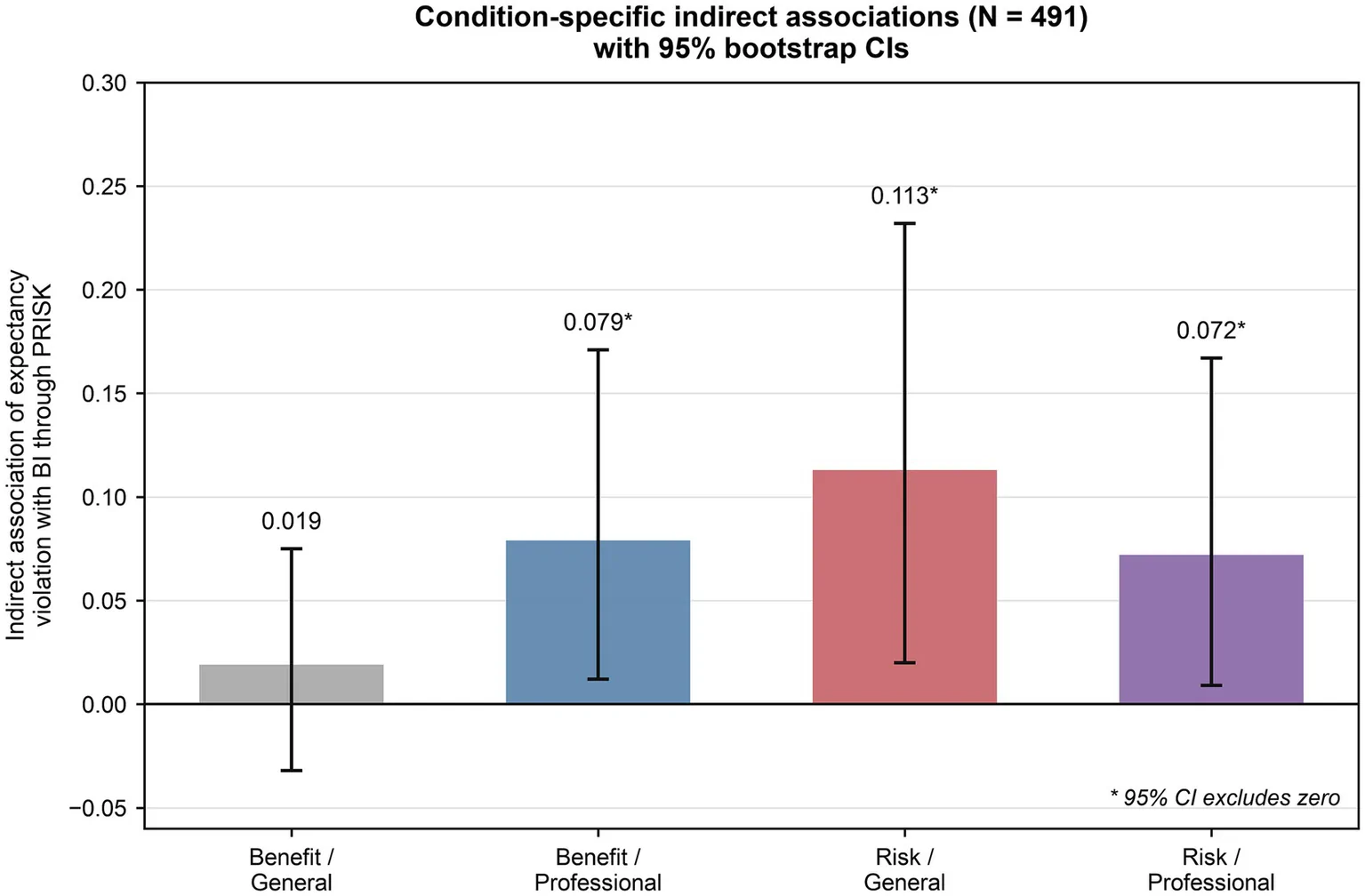
Condition-specific indirect associations of expectancy violation with BI through PRISK, with 95% bootstrap confidence intervals (*N* = 491). The omnibus three-way index included zero.

Three of the four condition-specific products excluded zero: benefit amplification with a professional model, effect = 0.079, 95% bootstrap CI [0.012, 0.171]; risk amplification with a general-purpose model, effect = 0.113, 95% CI [0.020, 0.232]; and risk amplification with a professional model, effect = 0.072, 95% CI [0.009, 0.167]. The benefit-amplification/general-model product was 0.019, 95% CI [−0.032, 0.075]. The findings were consistent with H8a in three configurations, but the pattern was not universal across conditions. The estimates are summarized in Table 4 and Figure 3.

**Table 4:** Condition-specific indirect associations of expectancy violation with BI through PRISK (*N* = 491).

Condition	Conditional indirect association	Bootstrap SE	95% CI LL	95% CI UL
Benefit amplification/General-purpose model	0.019	0.026	−0.032	0.075
Benefit amplification/Professional model	0.079	0.041	0.012	0.171
Risk amplification/General-purpose model	0.113	0.054	0.020	0.232
Risk amplification/Professional model	0.072	0.040	0.009	0.167

Media amplification was coded 0 = benefit amplification and 1 = risk amplification; model source was coded 0 = general-purpose model and 1 = professional model; expectancy violation was coded 0 = negative and 1 = positive. Positive products indicate that positive expectancy violation was statistically associated with stronger BI through lower PRISK. The omnibus three-way index included zero.

Only one of the six pairwise contrasts excluded zero: the risk-amplification/general-model product exceeded the benefit-amplification/general-model product, difference = 0.094, 95% CI [0.010, 0.225]. The simple index for media amplification within the general-model condition was 0.094, 95% CI [0.010, 0.225]. The corresponding index within the professional-model condition was −0.006, 95% CI [−0.085, 0.071]; the model-source indices were 0.060 within benefit amplification, 95% CI [−0.005, 0.168], and −0.040 within risk amplification, 95% CI [−0.134, 0.022]. The omnibus index, defined as the professional-model media-amplification difference minus the corresponding general-model difference, was −0.100, 95% CI [−0.260, 0.0003]. Because this interval included zero, H8b was not supported.

## 5 Discussion

This study examined how media amplification, model source, and expectancy violation shape prospective evaluation of GenAI in a health-consultation setting. Media amplification had its clearest effect on PRISK, while professional model source and positive expectancy violation showed favorable average effects on ATT, BI, and PRISK. The primary Type III models showed significant three-way interactions for all three outcomes. Sensitivity analyses provided the most consistent evidence for PRISK; the ATT and BI terms varied across specifications.

### 5.1 Conditional patterns in GenAI acceptance

GenAI acceptance in consequential settings reflects the configuration of positive and negative cues. Media amplification did not change ATT or BI on average even though it strongly increased PRISK. Positive expectancy violation produced a small, nonsignificant ATT contrast in the benefit-amplification/general-model condition but a substantially larger contrast in the risk-amplification/professional-model condition. Lower-order terms represent tendencies averaged across the third factor and are therefore interpreted together with the condition-specific estimates.

Rapid use can coexist with uneven trust: users may accept GenAI for particular tasks or cue configurations while withholding broader reliance. Trust research similarly shows that users may appreciate algorithmic assistance in some tasks while resisting it in domains where human uniqueness, accountability, or professional responsibility remains salient (Castelo et al., 2019; Longoni et al., 2019; Yalcin et al., 2022). This study extends that work by showing that, within a fixed health-consultation task, prospective evaluations of reliance vary with experimentally manipulated communication cues. Acceptance depends partly on how media messages frame the technology, how the model is labeled, and how prior users’ evaluations describe its performance relative to expectations.

### 5.2 Media amplification and risk salience: implications for SARF

The SARF-related findings place risk amplification primarily in risk appraisal. SARF describes how risk signals are selected, interpreted, and circulated, with consequences for risk perception and behavioral responses (Kasperson et al., 1988, 2022). In the present experiment, risk amplification clearly raised PRISK, while average ATT and BI remained statistically similar across amplification conditions.

The three-way terms suggest that media amplification can change the diagnostic value of model-source and expectancy-violation cues. Under risk amplification, professional-model cues and favorable prior-user evaluations may carry greater weight because they address salient concerns about harm.

Under benefit amplification, the positive expectancy-violation contrast was small for the general-purpose model. The professional-model condition showed a clearer increase in ATT and BI.

These findings connect the signal-selection and emphasis component of SARF to GenAI evaluation: the selective emphasis of benefits and risks shaped risk salience and the interpretation of later cues. Research on AI risk frames and expert amplification similarly identifies platform visibility, expert authority, and governance discourse as sources of salience before lay evaluation (Fjaeran et al., 2024; Schwarz, 2024).

### 5.3 Model-source credibility in AI-mediated evaluation

Classic credibility research shows that expertise and trustworthiness become influential when audiences cannot independently verify claims (Hovland and Weiss, 1951; Metzger et al., 2010; Pornpitakpan, 2004). That condition fits GenAI health consultation: most users cannot judge whether an answer is medically complete, appropriately cautious, or missing a warning sign. In this setting, a professional model cue gave participants a reason to see the system as more appropriate for the task.

GenAI outputs can sound polished even when the basis of the answer is uncertain. In a health-consultation scenario, users appear to assess whether a system fits the domain. The professional cue implied specialization and task fit, which helps explain the main effects on ATT, BI, and PRISK.

The professional-label advantage was largest when risk amplification was paired with positive expectancy violation, suggesting that the cue became more diagnostic when a favorable prior-user report addressed salient harm. This conditional pattern indicates that credibility in AI-mediated communication is provisional. A professional model cue supplies an initial claim of domain fit whose value is revised as users interpret the amplification context and prior-user evaluation (Lee, 2024).

### 5.4 Expectancy violation and perceived-risk associations: implications for EVT

Positive expectancy violation, conveyed through prior-user reports, improved ATT and BI and lowered PRISK on average. The findings concern prospective evaluation after better- or worse-than-expected information and show that its influence varied with media amplification and model source.

Positive and negative expectancy violations can have asymmetric implications in health communication. A negative report can make missed warnings, inappropriate reassurance, or unreliability more diagnostic because the potential cost of error is salient. A favorable report can improve evaluation and is weighed together with task appropriateness and source competence. The asymmetry is consistent with negativity-bias research and with evidence that responses to algorithmic performance vary by task and perceived consequence (Rozin and Royzman, 2001; Dietvorst et al., 2015; Logg et al., 2019).

Within EVT, the prior-user report supplied the expectancy violation, while model source supplied a basis for communicator reward valence. Favorable expectancy violation may carry greater weight when attached to a professionally positioned model or when it addresses risk made salient by amplification.

Prior human-AI studies link expectancy violation to competence judgments and continued engagement; the present pattern locates that appraisal within media-and source-defined contexts (Buder et al., 2024; Rheu et al., 2024).

Three condition-specific products through PRISK excluded zero. Because PRISK and BI were measured concurrently, these results are interpreted as conditional indirect associations. In most conditions, favorable prior-user reports coincided with both lower perceived risk and stronger intention, whereas the benefit-amplification/general-model condition showed little such association. This pattern is consistent with expectancy information being weighed as risk-relevant evidence.

The moderation evidence was selective. Only one of six contrasts among the four products and only one simple-index confidence interval excluded zero; the omnibus three-way index included zero.

### 5.5 Integrating SARF, source credibility theory, and EVT

The integrated account centers on cue dependence in prospective GenAI evaluation. Media emphasis establishes the interpretive conditions under which source identity and expectancy information acquire meaning. When risk is salient, users have stronger reason to attend to domain competence and to performance information that bears on possible harm. Under benefit amplification, favorable expectations may already be elevated, leaving less scope for an additional positive report from a general-purpose source. The significant three-way terms are consistent with this context-sensitive weighting of communication cues.

The conditional patterns further clarify how source credibility and expectancy violation work together. The professional label conveyed task fit, and its largest advantage emerged when risk amplification was paired with positive expectancy violation. In that configuration, the favorable prior-user report supported the competence implied by the professional positioning at a point when such competence was especially consequential. Positive expectancy violation had little additional influence in the benefit-amplification/general-model condition, where the available cues provided weaker support for domain-specific reassurance.

Perceived risk provides the clearest connection between this evaluative process and prospective acceptance. Risk amplification produced the largest average effect in the study, while favorable expectancy information frequently coincided with lower PRISK and stronger BI across the experimental configurations. Risk appraisal therefore appears to be the point at which media salience, perceived domain competence, and reported performance converge. Prospective acceptance reflects how well these cues fit together within the communication context in which the system is evaluated.

### 5.6 Communication, design, and governance implications

Expectation calibration is the clearest communication implication of the findings. Benefit-and risk-oriented messages establish different contexts for evaluating later source and performance cues. Communicators should connect claims about benefits and risks to concrete information about domain competence, uncertainty, and appropriate use. This gives users a basis for judging when reliance is warranted in a health-information task.

Professional positioning gains practical value when its basis is visible. Health-oriented systems can make specialization legible through evidence sources, uncertainty statements, and routes to human review. Studies of health chatbots and AI-generated health advice likewise show that cues of carefulness, contingency, and threat management influence user responses (Dong and Wu, 2025; Jin and Eastin, 2025; Qin et al., 2024). Bounded-use guidance and clear escalation advice can locate professional claims within the task and risk context.

Governance becomes communicatively relevant when safeguards are visible at the point of use. Use boundaries, traceability, and clear recourse can enter users’ risk assessments directly. This implication aligns with regulatory and sociotechnical research showing that deployment conditions shape how people encounter uncertainty and accountability (Dunn et al., 2023; Meskó and Topol, 2023; Dobbe and Wolters, 2024; Zanotti et al., 2024). Visible governance may therefore support more informed evaluation of GenAI health consultation.

### 5.7 Limitations

The study has six limitations. First, the vignette-based online experiment and self-reported intention measures were useful for isolating evaluative patterns, but future work should examine actual use, repeated interaction, and longer-term learning. Second, the findings concern general health consultation; diagnostic, treatment, or emergency contexts may yield different patterns. Third, the professional-model manipulation condensed several credibility signals into a single source cue. Real-world credibility also draws on performance, evidence, institutional backing, and visible governance. Fourth, the explicit manipulations helped ensure construct clarity but made the cues more salient than many real-world signals. Fifth, because perceived risk and behavioral intention were measured in the same post-exposure questionnaire, the secondary analysis supports only a conditional indirect association and cannot establish temporal order; longitudinal, panel, or experimental-causal-chain designs that directly manipulate perceived risk would be needed to test a causal pathway. Finally, the three-way interactions were small (partial η^2^ = 0.008–0.009); the perceived-risk pattern held across sensitivity specifications, whereas replication with larger samples would help establish the stability of the attitude and intention patterns.

## 6 Conclusion

This vignette-based health-consultation experiment shows that prospective acceptance of GenAI is shaped by the communication environment surrounding the system. Media amplification most clearly influenced perceived risk, while professional model-source cues and positive expectancy violation improved ATT and BI and reduced PRISK on average. The interaction patterns indicated that the meaning of source and expectancy cues varied across benefit-and risk-oriented contexts. Condition-specific indirect associations were evident in three configurations; the omnibus joint-moderation index did not support H8b.

These findings position media amplification as the setting in which audiences evaluate model identity and prior-user reports of better- or worse-than-expected performance. Media emphasis establishes which consequences are salient, source cues signal perceived task fit, and expectancy-relevant information informs judgments of likely performance. Perceived risk emerged as the clearest point of convergence among these cues.

For health communication, claims about GenAI should make their basis clear. Information about domain suitability, supporting evidence, and appropriate use may help users form proportionate expectations when considering GenAI for general health-information consultation.

## Data Availability

The raw data supporting the conclusions of this article will be made available by the authors, without undue reservation.
